# Association of SDF-1 and its receptor CXCR4 polymorphisms on the susceptibility of diabetic retinopathy in the Taiwanese population

**DOI:** 10.3389/fgene.2023.1296773

**Published:** 2023-11-23

**Authors:** Shu-Yen Peng, Chih-Chun Chuang, Yih-Shiou Hwang, Chieh-Hung Yen, Chia-Yi Lee, Shun-Fa Yang

**Affiliations:** ^1^ Institute of Medicine, Chung Shan Medical University, Taichung, Taiwan; ^2^ Department of Ophthalmology, Jen-Ai Hospital, Taichung, Taiwan; ^3^ Department of Ophthalmology, Changhua Christian Hospital, Changhua, Taiwan; ^4^ Department of Post-Baccalaureate Medicine, College of Medicine, National Chung Hsing University, Taichung, Taiwan; ^5^ Department of Ophthalmology, Chang Gung Memorial Hospital, Taoyuan, Taiwan; ^6^ College of Medicine, Chang Gung University, Taoyuan, Taiwan; ^7^ Department of Ophthalmology, Chang Gung Memorial Hospital, Xiamen Branch, Xiamen, China; ^8^ Graduate Institute of Biomedical Engineering, Chang Gung University, Taoyuan, Taiwan; ^9^ Department of Ophthalmology, Chang Gung Memorial Hospital at Linkou, Taoyuan, Taiwan; ^10^ Nobel Eye Institute, Taipei, Taiwan; ^11^ Department of Medical Research, Chung Shan Medical University Hospital, Taichung, Taiwan

**Keywords:** diabetic retinopathy, SDF-1, CXCR4, single-nucleotide polymorphisms, proliferative diabetic retinopathy

## Abstract

Stromal cell-derived factor-1 (SDF-1) and its receptor CXC chemokine 4 (CXCR4) have been demonstrated to play critical roles in diabetic retinopathy (DR). This study investigated whether single-nucleotide polymorphisms (SNPs) of SDF-1 and its receptor CXCR4 are correlated with diabetic retinopathy (DR). Three SDF-1 SNPs, namely, rs1801157 (G/A), rs2297630 (G/A), and rs266085 (T/C), and two CXCR4 SNPs, namely, rs2228014 (C/T) and rs6430612 (C/T), were chosen and genotyped via the TaqMan allelic discrimination for 454 non-DR subjects and 276 DR individuals. Our results revealed that subjects carrying SDF-1 SNP rs2297630 GA (AOR: 2.962, 95% CI: 1.279-6.861, *p* = 0.011) and SDF-1 SNP rs2297630 GA + AA (AOR: 3.095, 95% CI: 1.394-6.872, *p* = 0.006) had significantly higher risk in the non-proliferative diabetic retinopathy (NPDR) groups than in the non-DR group. Further analyses using the datasets from the Genotype-Tissue Expression (GTEx) Portal revealed that SDF-1 SNP rs2297630 GA and AA genotypic variants have higher SDF-1 expression than the GG wild-type alleles (*p* = 0.000016). In conclusion, our findings revealed that SDF-1 SNP rs2297630 was associated with NPDR.

## Introduction

Diabetic retinopathy (DR) is a specific microvascular complication in diabetes mellitus (DM) (Lin et al., 2021; Teo et al., 2021). Its global prevalence was estimated to be one-fifth among individuals with diabetes (Teo et al., 2021). The abnormalities of DR include microaneurysm, retinal hemorrhage, venous beading, exudates, cotton wool spots, intraretinal microvascular abnormalities, and neovascularization of the retina. Based on the presence of retinal neovascularization, DR could be divided into non-proliferative diabetic retinopathy (NPDR) and proliferative retinopathy (PDR). To treat visual-threatening DR, treatment options include laser photocoagulation, intravitreal injection of anti-vascular endothelial growth factors (anti-VEGF) or steroids, and vitrectomy. Without adequate treatment, DR may progress rapidly and cause visual impairment (Bakri et al., 2022).

There are some factors that affect the development and progression of DR. A higher glycated hemoglobin (HbA1c) level, a longer duration of diabetes mellitus, hypertension, and dyslipidemia are associated with diabetic retinopathy (Cheung et al., 2010; Lin et al., 2021). Moreover, genetic factors play an essential role in DR, and genetic studies show a racial variation in DR’s incidence (Simó-Servat et al., 2013; Cho and Sobrin, 2014; Mankoč Ramuš et al., 2021; Chuang et al., 2022a; Chuang et al., 2022b; Lee et al., 2022). Genome-wide analysis of DNA methylation in patients with type 1 diabetes mellitus found that this is associated with PDR (Agardh et al., 2015). According to the understanding of the pathogenesis of DR and PDR, several candidate genes were thought to be related to DR and PDR (Petrovic, 2013). The screening of single-nucleotide polymorphisms (SNPs) is useful to find the genetic risk factors for DR.

Stromal cell-derived factor-1 (SDF-1) is a chemokine, and CXC chemokine 4 (CXCR4) is the receptor of SDF-1 (Chang et al., 2009; Teng et al., 2009; Sadri et al., 2022). SDF-1 is expressed by the stromal cells and was initially isolated from the murine bone marrow (Guleng et al., 2005; Dar et al., 2006; Jin et al., 2006). In the human retina, the location of SDF-1 is mainly at the inner photoreceptor matrix and retinal pigment epithelial (RPE) cells, and the location of CXCR4 is mainly in the inner segment of photoreceptors (Bhutto et al., 2006). A higher level of vitreous SDF-1 was found in patients with proliferative diabetic retinopathy (Butler et al., 2005; Keles et al., 2021). Intravitreal injection of triamcinolone in patients with diabetic retinopathy decreased the level of vitreous SDF-1 (Butler et al., 2005). In human serum, the SDF-1 level was higher in patients with at least severe non-proliferative diabetic retinopathy than in patients with less severe diabetic retinopathy (Meleth et al., 2005). In one *in vitro* study, treatment with high glucose on human retinal vascular endothelial cells (hRVECs) increased the protein levels of SDF-1 and CXCR4, and the CXCR4 antagonist inhibited the expression levels of angiogenesis-associated proteins in hRVECs (Wu et al., 2019). Moreover, the polymorphism of the SDF-1 and CXCR4 gene are already known to be associated with the development of DR (Djuric et al., 2010; Gharibi et al., 2022). A previous study reported that homozygous carriers of SDF-1 3′A genotype are associated with the risk of PDR (Djuric et al., 2010). However, Gharibi et al. indicated no association among SDF-1 SNP rs1801157 and CXCR4 SNP rs2228014 polymorphisms and the risk of PDR in Iran population (Gharibi et al., 2022). Despite several studies having investigated the functional role of SDF-1 and CXCR4 SNPs in DR, the relationships between SDF-1 and CXCR4 SNPs and DR in the Taiwanese population have not been explored. The aim of this study was to investigate the potential association between the genetic polymorphisms of SDF-1 and CXCR4 and PDR in the Taiwanese population with type 2 DM.

## Materials and methods

### Ethical declarations

The procedures of this study adhered to the declaration of Helsinki in 1964 and its amendments. Our study was approved by the Institutional Review Boards of Chung Shan Medical University Hospital (project identification code: CS1-20048). Signed written informed consents were signed by all the participants of this study.

### Subject selection

This prospective case–control study was conducted in Chung Shan Medical University Hospital. A total of 730 individuals with diabetes mellitus from the department of endocrinology were enrolled in this study. According to the records of the ophthalmic department, the participants were divided into three categories by the Early Treatment Diabetic Retinopathy Study (ETDRS) diabetic retinopathy grading scale: the non-DR group, the NPDR group, and the PDR group, in which were 454, 165, and 111 participants, respectively (Kataoka et al., 2023). DR was regarded as at least one of the subsequent findings: microaneurysm, dot- or flame-shape intraretinal hemorrhage, venous beading, hard exudate, cotton-wool spot, or intraretinal microvascular abnormality. The PDR was defined as one of the following findings: neovascularization of the optic disc or the retina, neovascular glaucoma, vitreous hemorrhage, or tractional retinal detachment. The non-DR group, which was considered the control group, included individuals with diabetes mellitus without DR.

### Medical data and samples collection

We collected the medical records of these patients, including their age, age of onset of DM, gender, duration of diabetes, HbA1c, insulin treatment, body mass index, blood pressure, renal function, and lipid profiles. All participants received venous blood drawing for the investigation of SDF-1 and CXCR4 polymorphisms (Tee et al., 2012). The blood samples were stored in ethylenediaminetetraacetic acid-containing tubes and were then centrifuged and preserved in the laboratory refrigerator at −80 degrees Celsius. If the genomes of the blood samples were degraded before the analyses, they would be excluded from our study.

### DNA obtain and definition of SDF-1 and CXCR SNP with Real-Time PCR

Three SDF-1 SNPs [rs1801157 (G/A), rs2297630 (G/A), and rs266085 (T/C)] and two CXCR4 SNPs [rs2228014 (C/T) and rs6430612 (C/T)] were chosen for genetic analysis because of their tendency of malignancy, coronary disease, or hematological disease (Runmin et al., 2018; Li et al., 2020; Lin et al., 2022; Mohamed et al., 2022). Following the manufacturer’s instructions for DNA isolation, the genome DNA was extracted from the leukocytes in the blood samples by the QIAamp DNA kits (Qiagen, Valencia, Valencia, CA, United States) (Hsiao et al., 2010; Su et al., 2018). Then, the samples were stored in the refrigerator at around −20 degrees Celsius. The SDF-1 and CXCR4 SNPs were sequenced by the ABI StepOne Real-Time PCR System (Applied Biosystems, Foster City, California). We analyzed the findings of the SDF-1 and CXCR4 genetics polymorphism via SDS version 3.0 software (Applied Biosystems, Foster City, CA, United States).

### Statistical analysis

In our study, we used SAS version 9.4 (SAS Institute Inc, NC, United States) for analysis. We used descriptive analysis containing mean value, standard deviation (SD), and percentage to reveal the clinical and laboratory characteristics of the non-DR group, NPDR group, and PDR group. The independent t-test was used to compare the differences in both the clinical and laboratory data between the non-DR group and the NPDR group and between the non-DR group and the PDR group, respectively. Then, we used multiple logistic regression models after controlling for age, the duration of diabetes, HbA1c, insulin treatment, serum creatinine levels, glomerular filtration rate, and HDL cholesterol levels to estimate the adjusted odds ratio (AOR) and corresponding 95% confidence intervals (CI) of the six SNP distributions between the non-DR group and the DR group. We further searched the data that were related to the SNPs in a public database, the Genotype-Tissue Expression (GTEx) Portal, to analyze the genotype–phenotype correlation (https://gtexportal.org/home/). A *p* value of less than 0.05 was defined as statistically significant.

## Results

### Clinical manifestation of study participants

Table 1 summarizes the clinical and laboratory characteristics among the non-DR, NPDR, and PDR groups. The mean age was 60.22 ± 11.22 years old in the non-DR group, 62.29 ± 10.76 years old in the NPDR group, and 63.09 ± 10.70 years old in the PDR group. The NPDR group and PDR group had a longer duration of DM, higher HbA1c level, higher percentage of insulin treatment, higher serum creatinine, and lower glomerular filtration rate compared to the non-DR group (all *p* < 0.05). The NPDR group revealed a lower HDL cholesterol level compared to the non-DR group (*p* = 0.014).

**TABLE 1:** Clinical and laboratory characteristics of patients with diabetic retinopathy and no diabetic retinopathy.

Variable	No diabetic retinopathy (*N* = 454)	Non-proliferative diabetic retinopathy (*N* = 165)	Proliferative diabetic retinopathy (*N* = 111)	*p* value^a^	*p* value^b^
Age (years)	60.22 ± 11.22	62.29 ± 10.76	63.09 ± 10.70	0.041	0.015
Age of onset (years)	50.84 ± 10.77	51.22 ± 10.24	49.76 ± 12.31	0.694	0.358
Gender (man)[n (%)]	240 (52.9%)	98 (59.4%)	54 (48.6%)	0.149	0.426
Duration of diabetes (years)	9.38 ± 7.03	11.07 ± 6.60	13.33 ± 9.50	0.008	<0.001
HbA1c [% (mmol/mol)]	6.96 ± 0.99	7.47 ± 1.37	7.78 ± 1.48	<0.001	<0.001
Insulin treatment [n (%)]	105 (23.1%)	73 (44.2%)	56 (50.5%)	<0.001	<0.001
Body mass index [kg/m^2^]	26.15 ± 4.32	26.17 ± 4.31	25.72 ± 4.27	0.953	0.349
Systolic blood pressure [mmHg]	135.44 ± 15.32	138.36 ± 15.47	135.83 ± 19.84	0.037	0.821
Diastolic blood pressure [mm Hg]	76.44 ± 11.27	76.73 ± 10.77	74.25 ± 12.60	0.771	0.074
Serum creatinine [mg/dL]	0.89 ± 0.35	1.43 ± 1.45	1.73 ± 2.33	<0.001	<0.001
Glomerular filtration rate [ml/min]	78.44 ± 27.69	64.39 ± 34.54	60.46 ± 33.55	<0.001	<0.001
Total cholesterol [mmol/L]	160.52 ± 43.04	167.29 ± 45.83	161.77 ± 50.61	0.090	0.803
HDL cholesterol [μmol/L]	46.29 ± 12.64	43.42 ± 13.22	44.67 ± 14.24	0.014	0.269
LDL cholesterol [μmol/L]	86.60 ± 28.22	86.83 ± 29.28	85.53 ± 38.43	0.928	0.754
Triglycerides, [μmol/L]	140.19 ± 164.95	165.61 ± 122.92	141.48 ± 107.68	0.071	0.942

^a^ No Diabetic Retinopathy vs. Non-Proliferative Diabetic Retinopathy.

^b^ No Diabetic Retinopathy vs. Proliferative Diabetic Retinopathy.

### SDF-1 and CXCR4 SNPs distribution among different DR populations

In the comparison of the SDF-1 and CXCR4 SNPs between the non-DR and the DR group, the distribution frequency of the SDF-1 and CXCR4 SNPs was similar to that of wild type (all *p* > 0.05) (Table 2). Moreover, the distribution frequencies of the SDF-1 and CXCR4 SNPs between the non-DR, NPDR, and PDR groups were similar to that of the wild type (all *p* > 0.05) (Table 3; Table 4). Between the non-DR and the NPDR groups in the younger group (≤60 years), the results revealed that subjects carrying the SDF-1 SNP rs2297630 GA (AOR: 2.962, 95% CI: 1.279-6.861, *p* = 0.011) and SDF-1 SNP rs2297630 GA + AA (AOR: 3.095, 95% CI: 1.394-6.872, *p* = 0.006) had a significantly higher risk in the NPDR group than in the non-DR group (Table 5).

**TABLE 2:** Odds ratios (ORs) and 95% confidence intervals (CIs) of diabetic retinopathy associated with SDF-1α/CXCR4 axis genotypic frequencies.

Variable	No diabetic retinopathy (*N* = 454)	Diabetic retinopathy (*N* = 276)	AOR (95% CI)	*p* value
SDF-1				
rs1801157				
GG	215 (47.4%)	139 (50.4%)	1.000 (reference)	
GA	204 (44.9%)	119 (43.1%)	0.861 (0.605-1.225)	*p* = 0.406
AA	35 (7.7%)	18 (6.5%)	0.727 (0.369-1.431)	*p* = 0.356
GA + AA	239 (52.6%)	137 (49.6%)	0.840 (0.599-1.180)	*p* = 0.315
rs2297630				
GG	364 (80.2%)	212 (76.8%)	1.000 (reference)	
GA	80 (17.6%)	56 (20.3%)	1.138 (0.732-1.768)	*p* = 0.566
AA	10 (2.2%)	8 (2.9%)	1.688 (0.615-4.630)	*p* = 0.309
GA + AA	90 (19.8%)	64 (23.2%)	1.200 (0.792-1.817)	*p* = 0.390
rs266085				
TT	157 (34.6%)	87 (31.5%)	1.000 (reference)	
TC	223 (49.1%)	140 (50.7%)	0.943 (0.646-1.376)	*p* = 0.760
CC	74 (16.3%)	49 (17.8%)	1.040 (0.627-1.724)	*p* = 0.879
TC + CC	297 (65.4%)	189 (68.5%)	0.966 (0.675-1.382)	*p* = 0.851
CXCR4				
rs2228014				
CC	348 (76.7%)	208 (75.4%)	1.000 (reference)	
CT	94 (20.7%)	63 (22.8%)	1.071 (0.710-1.616)	*p* = 0.743
TT	12 (2.6%)	5 (1.8%)	0.755 (0.234-2.436)	*p* = 0.638
CT + TT	106 (23.3%)	68 (24.6%)	1.036 (0.697-1.540)	*p* = 0.860
rs6430612				
CC	413 (91.0%)	256 (92.8%)	1.000 (reference)	
CT	41 (9.0%)	20 (7.2%)	0.901 (0.488-1.663)	*p* = 0.738
TT	0 (0.0%)	0 (0.0%)	---	---
CT + TT	41 (9.0%)	20 (7.2%)	0.901 (0.488-1.663)	*p* = 0.738

The adjusted odds ratios (AORs) with their 95% confidence intervals were estimated by multiple logistic regression models after controlling for age, the duration of diabetes, HbA1c, insulin treatment, serum creatinine levels, glomerular filtration rate, and HDL cholesterol levels.

**TABLE 3:** Odds ratios (ORs) and 95% confidence intervals (CIs) of non-proliferative diabetic retinopathy associated with SDF-1α/CXCR4 axis genotypic frequencies.

Variable	No diabetic retinopathy (*N* = 454)	Non-proliferative diabetic retinopathy (*N* = 165)	AOR (95% CI)	*p* value
SDF-1				
rs1801157				
GG	215 (47.4%)	80 (48.5%)	1.000 (reference)	
GA	204 (44.9%)	73 (44.2%)	0.911 (0.608-1.364)	*p* = 0.651
AA	35 (7.7%)	12 (7.3%)	0.832 (0.388-1.782)	*p* = 0.635
GA + AA	239 (52.6%)	85 (51.5%)	0.899 (0.609-1.326)	*p* = 0.591
rs2297630				
GG	364 (80.2%)	125 (75.8%)	1.000 (reference)	
GA	80 (17.6%)	33 (20.0%)	1.242 (0.756-2.041)	*p* = 0.392
AA	10 (2.2%)	7 (4.2%)	2.479 (0.891-6.896)	*p* = 0.082
GA + AA	90 (19.8%)	40 (24.2%)	1.381 (0.871-2.189)	*p* = 0.170
rs266085				
TT	157 (34.6%)	58 (35.2%)	1.000 (reference)	
TC	223 (49.1%)	81 (49.1%)	0.825 (0.538-1.265)	*p* = 0.378
CC	74 (16.3%)	26 (15.8%)	0.809 (0.448-1.461)	*p* = 0.482
TC + CC	297 (65.4%)	107 (64.8%)	0.821 (0.548-1.231)	*p* = 0.340
CXCR4				
rs2228014				
CC	348 (76.7%)	128 (77.6%)	1.000 (reference)	
CT	94 (20.7%)	35 (21.2%)	1.133 (0.711-1.804)	*p* = 0.599
TT	12 (2.6%)	2 (1.2%)	0.395 (0.074-2.112)	*p* = 0.278
CT + TT	106 (23.3%)	37 (22.4%)	1.047 (0.665-1.648)	*p* = 0.843
rs6430612				
CC	413 (91.0%)	155 (93.9%)	1.000 (reference)	
CT	41 (9.0%)	10 (6.1%)	0.719 (0.340-1.518)	*p* = 0.387
TT	0 (0.0%)	0 (0.0%)	---	---
CT + TT	41 (9.0%)	10 (6.1%)	0.719 (0.340-1.518)	*p* = 0.387

The adjusted odds ratios (AORs) with their 95% confidence intervals were estimated by multiple logistic regression models after controlling for age, the duration of diabetes, HbA1c, insulin treatment, serum creatinine levels, glomerular filtration rate, and HDL, cholesterol levels.

**TABLE 4:** Odds ratios (ORs) and 95% confidence intervals (CIs) of proliferative diabetic retinopathy associated with SDF-1α/CXCR4 axis genotypic frequencies.

Variable	No diabetic retinopathy (*N* = 454)	Proliferative diabetic retinopathy (*N* = 111)	AOR (95% CI)	*p* value
SDF-1				
rs1801157				
GG	215 (47.4%)	59 (53.2%)	1.000 (reference)	
GA	204 (44.9%)	46 (41.4%)	0.712 (0.419-1.209)	*p* = 0.209
AA	35 (7.7%)	6 (5.4%)	0.539 (0.189-1.539)	*p* = 0.249
GA + AA	239 (52.6%)	52 (46.8%)	0.682 (0.410-1.135)	*p* = 0.141
rs2297630				
GG	364 (80.2%)	87 (78.4%)	1.000 (reference)	
GA	80 (17.6%)	23 (20.7%)	0.853 (0.433-1.680)	*p* = 0.646
AA	10 (2.2%)	1 (0.9%)	0.366 (0.032-4.186)	*p* = 0.419
GA + AA	90 (19.8%)	24 (21.6%)	0.801 (0.414-1.552)	*p* = 0.511
rs266085				
TT	157 (34.6%)	29 (26.1%)	1.000 (reference)	
TC	223 (49.1%)	59 (53.2%)	1.228 (0.684-2.206)	*p* = 0.492
CC	74 (16.3%)	23 (20.7%)	1.545 (0.740-3.225)	*p* = 0.247
TC + CC	297 (65.4%)	82 (73.9%)	1.305 (0.749-2.275)	*p* = 0.348
CXCR4				
rs2228014				
CC	348 (76.7%)	80 (72.1%)	1.000 (reference)	
CT	94 (20.7%)	28 (25.2%)	0.906 (0.483-1.699)	*p* = 0.759
TT	12 (2.6%)	3 (2.7%)	1.971 (0.505-7.691)	*p* = 0.329
CT + TT	106 (23.3%)	31 (27.9%)	1.001 (0.555-1.802)	*p* = 0.998
rs6430612				
CC	413 (91.0%)	101 (91.0%)	1.000 (reference)	
CT	41 (9.0%)	10 (9.0%)	1.372 (0.595-3.162)	*p* = 0.458
TT	0 (0.0%)	0 (0.0%)	---	---
CT + TT	41 (9.0%)	10 (9.0%)	1.372 (0.595-3.162)	*p* = 0.458

The adjusted odds ratios (AORs) with their 95% confidence intervals were estimated by multiple logistic regression models after controlling for age, the duration of diabetes, HbA1c, insulin treatment, serum creatinine levels, glomerular filtration rate, and HDL cholesterol levels.

**TABLE 5:** Odds ratios (ORs) and 95% confidence intervals (CIs) of diabetic retinopathy associated with SDF-1α/CXCR4 axis genotypic frequencies in age ≤60.

Variable	No diabetic retinopathy (N = 192)	Non-proliferative diabetic retinopathy (*N* = 66)	AOR (95% CI)	*p* value
SDF-1				
rs1801157				
GG	95 (49.5%)	34 (51.5%)	1.000 (reference)	
GA	83 (43.2%)	29 (43.9%)	0.871 (0.436-1.740)	*p* = 0.695
AA	14 (7.3%)	3 (4.6%)	0.235 (0.043-1.272)	*p* = 0.093
GA + AA	97 (50.5%)	32 (48.5%)	0.750 (0.383-1.472)	*p* = 0.404
rs2297630				
GG	159 (82.8%)	47 (71.2%)	1.000 (reference)	
GA	29 (15.1%)	16 (24.2%)	2.962 (1.279-6.861)	*p* = 0.011*
AA	4 (2.1%)	3 (4.6%)	4.043 (0.693-23.591)	*p* = 0.121
GA + AA	33 (17.2%)	19 (28.8%)	3.095 (1.394-6.872)	*p* = 0.006*
rs266085				
TT	66 (34.4%)	22 (33.3%)	1.000 (reference)	
TC	97 (50.5%)	32 (48.5%)	1.136 (0.535-2.412)	*p* = 0.739
CC	29 (15.1%)	12 (18.2%)	2.029 (0.743-5.539)	*p* = 0.167
TC + CC	126 (65.6%)	44 (66.7%)	1.299 (0.637-2.647)	*p* = 0.472
CXCR4				
rs2228014				
CC	157 (81.8%)	52 (78.8%)	1.000 (reference)	
CT	32 (16.7%)	13 (19.7%)	1.650 (0.731-3.722)	*p* = 0.228
TT	3 (1.6%)	1 (1.5%)	0.331 (0.012-9.498)	*p* = 0.519
CT + TT	35 (18.2%)	14 (21.2%)	1.510 (0.679-3.355)	*p* = 0.312
rs6430612				
CC	172 (89.6%)	62 (93.9%)	1.000 (reference)	
CT	20 (10.4%)	4 (6.1%)	0.715 (0.219-2.341)	*p* = 0.580
TT	0 (0.0%)	0 (0.0%)	---	---
CT + TT	20 (10.4%)	4 (6.1%)	0.715 (0.219-2.341)	*p* = 0.580

The adjusted odds ratios (AORs) with their 95% confidence intervals were estimated by multiple logistic regression models after controlling for the duration of diabetes, HbA1c, insulin treatment, serum creatinine levels, glomerular filtration rate, and HDL, cholesterol levels. * *p* < 0.05.

### The correlations of SDF-1 SNP rs2297630 with SDF-1 expression levels

**FIGURE 1:**
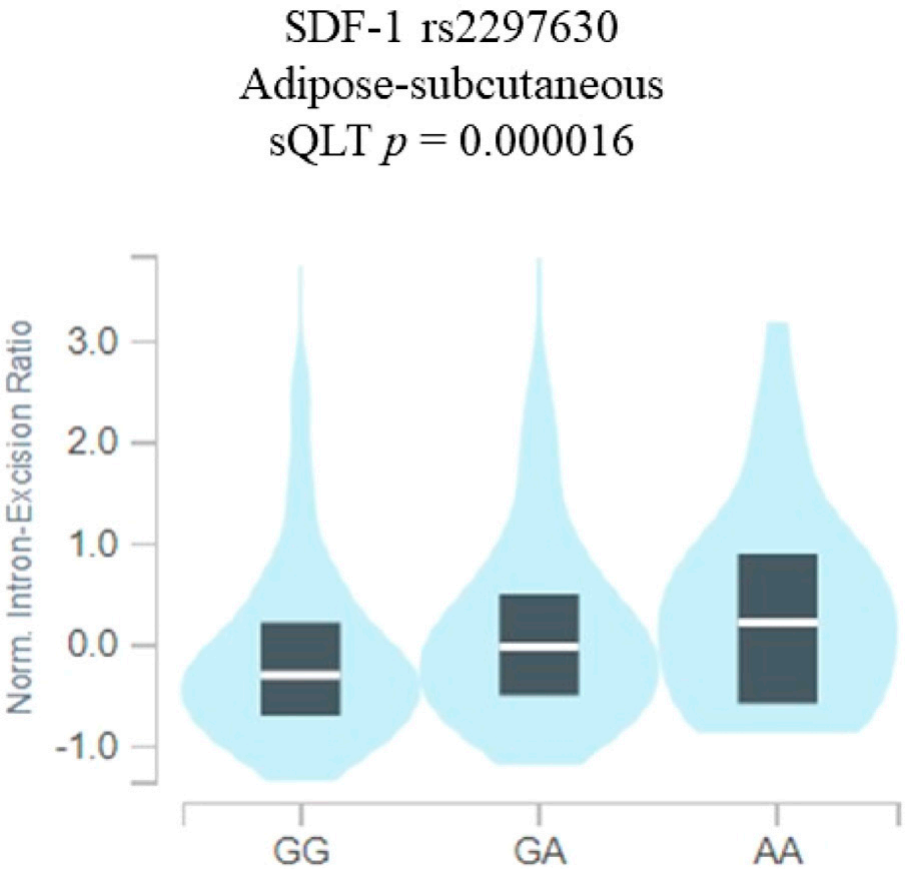
SDF-1 SNP rs2297630 regulates the expression of SDF-1. eQTL analysis of SDF-1 SNP rs2297630 in adipose subcutaneous tissues based on data from the GTEx portal. *p* values are calculated with the linear regression model.

Since the SDF-1 SNP rs2297630 was found to be associated with the NPDR groups, additional analyses using public datasets were performed to increase its putative functional relevance. By using the Genotype-Tissue Expression (GTEx) Portal, the results revealed that the SDF-1 SNP rs2297630 GA and AA genotypic variants have higher SDF-1 expression than the GG wild-type alleles (*p* = 0.000016) (Figure 1).

## Discussion

Our study demonstrated that rs2297630 of the SDF-1 gene was associated with NPDR. The SDF-1 and CXCR4 signaling pathway was considered to have roles in angiogenesis, tumor growth, embryogenesis, and wound healing (Sadri et al., 2022). A previous study showed that SDF-1 bound to its receptor CXCR4 on the endothelial cells and induced angiogenesis by releasing vascular endothelial growth factors (VEGF) (Mirshahi et al., 2000). VEGF was reported to induce expression of CXCR4 on the endothelial cells (Salcedo et al., 1999). The signaling pathway was also related to breast cancer, prostate cancer, lung cancer, leukemia, and pancreatic cancer (Ueda et al., 2006; Juarez et al., 2007; Nervi et al., 2009; Gao et al., 2010; Shi et al., 2020; Wang et al., 2021; Luo et al., 2022). Many SDF-1 and CXCR4 SNPs were found to be related to different tumors.

The genetic factors, linkage analyses, candidate gene studies, and genome-wide association studies (GWAS) were found to be correlated with DR risk or progression (Cabrera et al., 2020; Forrest et al., 2021). In a genome-wide polygenic risk score study, Forrest et al. established the significant polygenic underpinnings of DR (Forrest et al., 2021). Moreover, in a genome-wide association study in 286 Mexican Americans with type 2 diabetes, Fu et al. reported that CAMK4 (calcium/calmodulin-dependent protein kinase IV) rs2300782 and FMN1 (formin 1) gene SNP rs10519765 were associated with severe diabetic retinopathy (Fu et al., 2010). In a systematic meta-analysis study, Abhary et al. concluded that variations within the aldose reductase gene are highly significantly associated with DR development (Abhary et al., 2009). Therefore, the screening of SNPs is useful to discover the genetic risk factors of DR.

The current study revealed that the distribution frequency of SDF-1 SNP rs2297630 GA + AA is significantly higher in NPDR patients than non-DR patients. The rs2297630 SNP is located in intron 3, +8906 of the SDF-1 gene (Xiao et al., 2008; Ku et al., 2013). Individuals with rs2297630 AA genotype were reported to have higher SDF-1 levels and a lower circulating endothelial progenitor cells number (Xiao et al., 2008). Our results also revealed that the SDF-1 SNP rs2297630 GA and AA genotypes have higher SDF-1 expression than the GG wild-type alleles (Figure 1). Xiao et al. reported that this SDF-1 SNP rs2297630 can affect SDF-1 expression or mRNA splicing to alter SDF-1 expression level (Xiao et al., 2008; Lin et al., 2022). Moreover, the rs2297630 polymorphism was found to be significantly linked to many diseases (Yin et al., 2019; Ji et al., 2023; Wang et al., 2023). The G allele of rs2297630 in SDF-1 was found to be associated with a higher risk of hypertension (Wang et al., 2023). The A allele of SDF-1 SNP rs2297630 was found to be a risk factor for pediatric acute lymphoblastic lymphoma (Ji et al., 2023). Rs2297630 AA genotype was reported to increase the risk of type 2 diabetes mellitus development (Yin et al., 2019). The A allele of rs2297630 was related to the increased production of interleukin 6 (Wang et al., 2019), which was thought to be associated with not only the occurrence of sepsis but also with diabetes mellitus and diabetic retinopathy (Mocan et al., 2006; Chen et al., 2017; Akbari and Hassan-Zadeh, 2018; Yao et al., 2019).

There are some limitations in our study. First, the case numbers in some subgroups were too low to be calculated adequately and may have led to statistical bias. Second, the diagnosis and staging of DR were conducted by different ophthalmologists in our hospital. Although our doctors used the same diagnostic criteria of non-DR, NPDR, and PDR, the clinical judgement may have varied. Third, the longitudinal effect of SDF-1 and CXCR4 SNPs could not be evaluated because of the case–control design of this study.

In conclusion, the distribution frequency of SDF-1 SNP rs2297630 GA + AA is more prevalent in NPDR patients than non-DR patients. Genetic analysis of the SDF-1 polymorphism may be suggested for DR individuals. Larger prospective studies to evaluate the association between the SDF-1 and CXCR4 polymorphism and the treatment outcome are necessary.

## Data Availability

The original contributions presented in the study are included in the article/Supplementary Material, further inquiries can be directed to the corresponding author.
